# Evolution in Response to Management Increases Invasiveness Among Experimental Populations of Duckweed ( *Lemna minor* )

**DOI:** 10.1111/eva.70060

**Published:** 2024-12-26

**Authors:** Taylor A. Zallek, Martin M. Turcotte

**Affiliations:** ^1^ Department of Biological Sciences University of Pittsburgh Pittsburgh Pennsylvania USA

**Keywords:** duckweed, experimental evolution, invasiveness, *Lemna minor*, pest management

## Abstract

Numerous management methods are deployed to try to mitigate the destructive impact of weedy and invasive populations. Yet, such management practices may cause these populations to inadvertently evolve in ways that have consequence on their invasiveness. To test this idea, we conducted a two‐step field mesocosm experiment; we evolved genetically diverse populations of the duckweed *Lemna minor* to targeted removal management and then tested the impact of that evolution in replicated invasions into experimental resident communities. We found that evolution in response to management increased invasiveness compared to populations evolved without management. This evolution in response to management had little effect on the impact of the invader on the resident species. These results illustrate the potential eco‐evolutionary consequences of management practices. Mitigating evolution to physical removal, in addition to pesticides, may be important to the long‐term success of integrated pest management.

## 1 Introduction

A population's ability to successfully establish following introductions at low abundance and invade recipient communities (i.e., invasiveness) is determined by many interacting factors including abiotic conditions, introduction frequency and abundance (i.e., propagule pressure), the genetic and functional diversity of both the invading and resident populations, and the species diversity of the resident community (Kleunen, Weber, and Fischer 2010; Blackburn et al. 2011; Colautti and Lau 2015; Catford et al. 2019). Invasiveness is a fundamental and variable characteristic of populations with important implications for species coexistence, community assembly, and succession (Shea and Chesson 2002; Fargione, Brown, and Tilman 2003; Prach and Walker 2011; Grainger, Levine, and Gilbert 2019). Moreover, invasiveness has applied importance in dictating the spread and appropriate management approaches of weedy or invasive populations. Predicting which species will spread and effectively managing their ecological and economic impact can also be further complicated by the fact that the properties of these species can change rapidly as the result of multiple evolutionary processes (Lee 2002; Dlugosch and Parker 2008; Colautti et al. 2017). Therefore, understanding both the ecological and evolutionary mechanisms behind these changes may be required to properly predict and manage population invasiveness and their impact (Stockwell, Hendry, and Kinnison 2003; Prentis et al. 2008; Phillips 2015).

Introduced or weedy populations can evolve rapidly during their spread to changing environmental conditions (Colautti and Barrett 2013; Moran and Alexander 2014; van Boheemen, Atwater, and Hodgins 2019), changes in species interactions (Lankau et al. 2009; Seifert, Bever, and Maron 2009), and through selection imposed by the process of spreading itself (Phillips, Brown, and Shine 2010; Shine, Brown, and Phillips 2011). There is growing evidence that such rapid evolution can alter range dynamics (Williams, Hufbauer, and Miller 2019; Miller et al. 2020) and interactions with recipient communities (Lankau 2012). In addition to evolving in response to novel abiotic and biotic conditions, weedy or invasive populations can also evolve in response to selection imposed by humans. As their populations increase in size and range, weedy or invasive species can threaten recipient populations or communities, environments, and economies and therefore become increasingly likely targets for human intervention (i.e., management). Management, or the repeated removal of individuals to reduce a harmful population's impact, can take many forms including biological, chemical, cultural, mechanical, and manual control. These management strategies can pose strong selective pressures resulting in rapid evolutionary responses by targeted populations (Turcotte et al. 2017). Resistance evolution in response to pesticides is a common and, often now, an expected response (Georghiou 1972; Hawkins et al. 2019; Kreiner et al. 2022). For example, multiple populations of the invasive aquatic plant *Hydrilla verticillata* have independently evolved resistance to the commonly applied herbicide Fluridone in the state of Florida, USA (Michel et al. 2004). After dramatically expanding their range and invasion into novel agricultural environments, native populations of common waterhemp ( *Amaranthus tuberculatus* ) have rapidly responded to increased management with the herbicide glyphosate by repeatedly evolving resistance (Kreiner et al. 2022).

In addition to the selection for herbicide‐resistant alleles, increased external sources of mortality can select for more rapid life‐history traits as seen in Trinidadian guppies (Reznick, Bryga, and Endler 1990) and also increased population growth rates in *Drosophila* (Stearns et al. 2000). Changes in population growth rate could directly lead to more abundant populations and faster spread which may cause stronger impacts on residents and communities. Yet, the opposite is also possible if traits providing a fitness advantage under management trade‐off with (or have neutral effects on interspecific competitive sensitivity) their ability to impact residents (Coomes and Grubb 2003; Gagneux et al. 2006; Ricciardi and Cohen 2007). While these (and other) examples of evolution in response to management appear to be adaptive under specific management regimes, determining whether this evolution alters a population's invasiveness remains generally unknown and undertested.

Experimental evolution can be used to move beyond observational inference to more robustly test how evolution impacts the ecological dynamics of populations (Fussmann, Loreau, and Abrams 2007; terHorst 2010; Turcotte, Reznick, and Hare 2011). One can thus compare the invasiveness of populations evolved with and without management or in contrast to an unevolved ancestral population. This approach has several advantages and can complement other types of observational and empirical research. Observational studies that only measure the evolution of allele and trait frequencies may overlook instances where this evolution has too small of an impact to matter ecologically. While several studies have utilized experimental evolution to understand eco‐evolutionary aspects related to range expansion and population spread (Fronhofer and Altermatt 2015; Williams, Kendall, and Levine 2016; Fronhofer, Gut, and Altermatt 2017; Ochocki and Miller 2017; Szűcs et al. 2017; Weiss‐Lehman, Hufbauer, and Melbourne 2017; Lustenhouwer, Williams, and Levine 2019), fewer studies have integrated experimental communities in tests of invasiveness (but see Faillace and Morin 2016, 2020; Saarinen, Lindström, and Ketola 2019). Whether the impact of evolution on invasiveness remains consistent in more natural conditions outside of the lab with many more selective pressures and sources of ecological variation remains untested to our knowledge.

The aquatic floating plant family of duckweed (Lemnaceae) are an excellent system to conduct ecological and evolutionary experiments both within and outside the lab (Landolt 1986; Laird and Barks 2018; Jewell and Bell 2022) and are increasingly being used in experimental evolution studies (Hart, Turcotte, and Levine 2019; Xu et al. 2019; Sandler et al. 2020; Tan, Kerstetter, and Turcotte 2021). Their small size and rapid asexual reproduction within 3–4 days (Ziegler et al. 2015) allow for multigenerational experiments featuring rapid evolutionary and population dynamics (Armitage and Jones 2019; Hess et al. 2022; Anneberg et al. 2023; Usui and Angert 2024). Duckweed also have extensive species and genotypic variation in numerous ecologically relevant phenotypic traits (Appenroth and Adamec 2015; Hitsman and Simons 2020). In addition, duckweed possess many similar characteristics to weedy and invasive plant populations including rapid growth, global species distributions, reliance on clonal reproduction, tolerance to many ecological conditions, and the ability to dominate local communities (Wang 1990; Scheffer et al. 2003; Ziegler et al. 2015; Ekperusi, Sikoki, and Nwachukwu 2019). Given that the majority of the worst invasive alien species globally are plants (Luque et al. 2014; Simberloff and Rejmanek 2019), having a representative model system is useful for making generalizable conclusions under similar ecological conditions and evolutionary constraints. Furthermore, duckweed offer the ability to conduct experimental evolution in outdoor mesocosm settings beyond tightly controlled laboratory conditions.

To test how evolution in response to management impacts population invasiveness, we conducted a multi‐phase outdoor mesocosm experiment. We first allowed replicated populations to evolve to managed (repeated removal of a large portion of the population) or unmanaged conditions for several generations. Following a common garden phase, we conducted an invasion assay into experimental communities to address the following questions: (1) Do evolved populations differ in their ability to establish and grow within a community? (2) Has evolution in response to management altered the impact of the invader on the residents and community? To further assess how establishment may differ with invader evolution, we also manipulated initial propagule size in the invasion assay and asked (3) Does the impact of evolution depend on propagule size?

## 2 Methods

### 2.1 Study System

In this experiment we used the common duckweed *Lemna minor* (L.) as a model to experimentally investigate the impact of evolution on invasiveness. This duckweed and the other community members, including *Spirodela polyrhiza* (L.), *Lemna trisulca* (L.), and *Wolffia spp*. (Horkel ex Schleid.) used in this experiment are all native to western Pennsylvania, USA (Block and Rhoads 2011). *Lemna minor* is a cosmopolitan, globally occurring plant species but other members of the genus (including *Lemna minuta* Kunth) have invaded European waterbodies (Ceschin et al. 2016). We did not use an invasive aquatic plant for several reasons. First, ethically we did not want to be responsible for the evolution or accidental introduction of non‐native and potentially invasive populations escaping our field mesocosms. Second, to conduct our evolution experiment we needed several genotypes of this species with characterized genetic markers, which could be challenging using invasive populations that have either recently passed through a genetic bottleneck or had previously experienced evolution to the same selective forces we are imposing. Instead, we wanted to observe the evolutionary emergence of weedy or invasive traits and properties from an otherwise benign species in this community context. Third, the community of duckweed we sampled as our “residents” had very few *L. minor* (less than 5% of total community biomass), as observed over multiple years (“Zallek” *unpublished data*).

*Lemna minor* was collected from different sites across western Pennsylvania in 2018–2020 (Table S1). Twelve genotypes were identified using 11 polymorphic microsatellite markers (Kerstetter et al. 2023) and each was maintained in separate lab colonies, in which they reproduce clonally, in nutrient media (Appenroth, Teller, and Horn 1996), modified by increasing KH_2_PO_4_ to obtain a 14:1 N:P ratio. The resident community was sampled from a separate site; Pennsylvania State Gamelands 151 (41.106241, −80.134670) on June 1, 2021. All duckweed were then transferred to the University of Pittsburgh's Pymatuning Lab of Ecology. *Lemna minor* and the mixed community of field‐collected species were maintained under semi‐natural outdoor mesocosm conditions in 10% media for 26 days. *Lemna minor* genotypes were maintained within plastic deli containers (approximately 950 mL, Item # 128HD32COMBO, WebstaurantStore, USA) and suspended on a floating raft made from foam insulation boards and placed within a 1135 L cattle tank which helped with temperature regulation. Cattle tanks were covered in two layers of 50% shade cloth (Green‐Tek #127616, Gemplers, USA). Residents were maintained within 189 L cattle tanks covered in 70% shade cloth (Green‐Tek #145666, Gemplers, USA) at densities comparable to those found in the wild. Water levels were consistently replenished, and media was supplemented every 3 weeks to maintain consistent concentrations. We carefully removed any *L. minor* from the field‐collected community prior to their experimental establishment. We then conducted a three‐part study consisting of experimental evolution, common garden, and an invasion experiment.

### 2.2 Evolution Experiment

Forty populations of *L. minor* were established on June 24, 2021, consisting of equal frequencies of 12 genotypes totaling 144 individuals per population in the same deli containers, rafts, and cattle tanks as described above. Treatment replicates were distributed evenly among tanks and randomized within them. Each tank was covered in two layers of 50% shade cloth. *Lemna minor* populations were allowed to grow over 11 weeks (approximately 13–19 generations) and could evolve through changes in genotypic frequencies (Hart, Turcotte, and Levine 2019). At three time points during the experiment (July 15, August 5, August 26), media was replaced in all containers and populations were photographed to estimate population size. At these same time points, we also simulated management in half the populations, culling these populations back to their initial number of 144 individuals. Individuals were randomly removed independent of any visible phenotypic traits. We did so by moving populations into larger containers before mixing and scattering individuals evenly across the water's surface. Then we collected individuals every few centimeters along a “Z‐pattern” until we had 144 to place back into their containers.

Our managed treatment‐induced selection was through a combination of three rounds of random reductions in population size followed by periods of growth between removal events. Together this created selection for traits that increased numeric abundance before removal such as higher reproductive rate and survival while also imposing genetic drift. Managed populations were transferred using plastic utensils so as not to damage fronds. “Non‐managed” populations were not culled but were similarly transferred to new containers and stirred using the same plastic utensils. Throughout the evolution phase of the experiment, six of the managed and five of the non‐managed populations were lost due to severe insect herbivory. To our knowledge, no insects were found in any of the remaining 29 populations. To prevent further herbivory, white sheer voile chiffon fabric covers were added following the first management phase (Sedona Designz, USA). During this time, the resident communities of the other three species were maintained in large outdoor tanks under the same media and shading conditions. The 12 isolated genotypes of *L. minor* used were maintained during the evolution experiment in similar conditions as described above. Nutrients were replaced at identical time periods and while populations weren't reduced to their initial founding population sizes, they were reduced to an intermediate amount (relative to managed and non‐managed populations) to maintain growing populations.

### 2.3 Common Garden

Following the evolution experiment, we transferred plants into common garden environments to minimize the impact of maternal effects and phenotypic plasticity. To avoid density effects, we only transferred a random sample of 240 individuals per population each into a new container with 10% media floating in a tank. This common garden period began on September 9, 2021, and ended after 1 week on September 16, 2021 (approximately two generations). The 12 *L. minor* genotypes were similarly transferred into the common garden conditions as the evolved populations. These genotypes were used to create non‐evolving controls (ancestral reconstruction) that provide insight into the directionality of evolution.

### 2.4 Invasion Experiment

On September 16, we established resident communities and added in a relatively small number of *L. minor* and tracked its invasion dynamics over 2 weeks. Duckweed communities growing in tanks were thoroughly mixed, and approximately 36 cm^2^ surface area was sampled and placed into a bottle, representing several hundred *S. polyrhiza, L. trisulca*, and *Wolffia* spp. individuals per species. These HDPE bottles were 6.5 cm diameter and 15.7 cm tall with the top section removed creating cylinders with one open end (Part # 30WAQ5, CaryCompany, USA) to which 250 mL of 10% media was added. This sub‐sampling method was designed to establish communities with high densities similar to those found in nature. Note that, while we may have only sampled 36 cm^2^ from the community, this did not reflect the total area (measured at the conclusion of the experiment) as duckweed are prone to overlapping vertically on the water's surface. These bottles were suspended in water‐filled 1250 L cattle tanks using dishwasher racks (Noble Products, 49‐compartment glass rack, Item#: 274RK491, WebstaurantStore, USA) covered in white chiffon. Tanks were then covered with 2 layers of 50% shade cloth. In each bottle we added, each bottle was then photographed and its initial total area was quantified.

We then initiated the invasion by adding *L. minor* at different initial propagule sizes of 12, 24, 48, or 96 individuals. Even the largest propagule size consisted of less than 5% of the initial biomass and surface area of each community and was therefore considered rare. Propagule size was crossed with the three evolutionary histories: populations that evolved with and without management as well as a “non‐evolved” control population. For these controls, we recreated the initial equal frequency of genotypes using the single genotype containers. For the low propagule size of 12, we used random pairs of six genotypes (each replicate was different) because we did not want to damage individuals by separating them from their clusters. The non‐evolving controls were replicated 10 times per propagule size. Each replicate of the evolving populations (14 for managed and 15 for non‐managed) was thus distributed into each propagule pressure treatment. Even numbers of containers representing each treatment were spread among the tanks. Containers with source populations from the same evolution container were all represented within the same tank.

The invasion experiment lasted 2 weeks, ending on September 30, 2021. The *L. minor* populations were then separated from their communities and photographed to estimate population size and surface area before collecting their dry biomass. Population sizes were estimated by averaging the final population number counted by 2–3 research assistants. Surface area was collected using Fiji/ImageJ (Schindelin et al. 2012) and averaged in similar fashion. Dry biomass was measured using pre‐weighed foil and oven drying them at 55°C for 72 h. Resident data (including surface area and dry biomass) were collected in a similar fashion as invader data described above but logistical constraints prevented us from quantifying species‐specific data.

Finally, to obtain additional insight into how management impacts duckweed evolution, we also grew one replicate per remaining evolution treatment (14 managed, 15 non‐managed, 10 non‐evolved) without resident species in the same conditions as above but only starting them at 24 individuals. Any remaining duckweed used in the experiment were destroyed to prevent the accidental release of genotypes or populations into ecosystems they weren't previously found.

### 2.5 Statistical Analyses

To test how experimental evolutionary history and propagule pressure interact to impact invasiveness, we performed a series of linear mixed effects models using R software and the package “nlme” (Pinheiro et al. 2023). Fixed effects included evolutionary treatment (managed, non‐managed, and non‐evolved), initial propagule size as a covariate, and their interaction as well as source container (i.e., the source population of the duckweed from the evolution experiment) as a random effect. For invasiveness, we began by combining multiple dependent factors including log transformed final abundance, log biomass (mg), and log surface area (mm^2^) of invaders by first performing a principal component analysis using the princomp function in R (R Core Team 2021). This was conducted to minimize multiple testing bias. We then tested each factor independently. These variables were log transformed to minimize the impact of heteroskedasticity. A constant value of 1 was added to all final biomass values before log transforming the data due to the existence of values less than 1. We also tested per capita biomass and per capita surface area. For the impact on residents, our response variables were resident biomass and surface area. We also measured total community biomass and surface area. To calculate the differences among groups, we used the Tukey method for comparing a family of three estimates implemented using the emmeans and emtrends functions in the “emmeans” package (Lenth et al. 2023).

## 3 Results

### 3.1 *L. minor* Growth Without Community

Before testing the impact of management evolution on invasion dynamics, we tested how managed, non‐managed, and non‐evolved populations grew alone without interspecific community competitors. Starting with an initial abundance of 24 individuals, these populations grew to be significantly different in final abundance (*F*_2,35_ = 4.259, *p* = 0.0221), biomass (*F*_2,34_ = 6.556, *p* = 0.0039), and surface area (*F*_2,35_ = 6.739, *p* = 0.0033). Specifically, we found that populations that evolved under management grew to significantly higher final abundances (+37%, *p* = 0.0264), biomass (+64%, *p* = 0.0039), and surface area (+93%, *p* = 0.0033) than the unmanaged evolving populations (Figure S1).

Individuals in these populations exhibited significant differences in their per capita surface area (*F*_2,35_ = 7.349, *p* = 0.0022, Figure S2) but not biomass (*F*_2,34_ = 1.365, *p* = 0.2691). Individuals in managed populations grew significantly greater per capita surface area relative to non‐managed (+38%, *p* = 0.0021, Figure S2) and non‐evolved (+29%, *p* = 0.0367) populations. There was no significant difference between non‐managed and non‐evolved per capita surface area (*p* = 0.8016). Next, we tested how evolution impacts the ability to invade a resident community (i.e., invasiveness).

### 3.2 Evolution's Impact on *L. minor* Invasiveness

**FIGURE 1:**
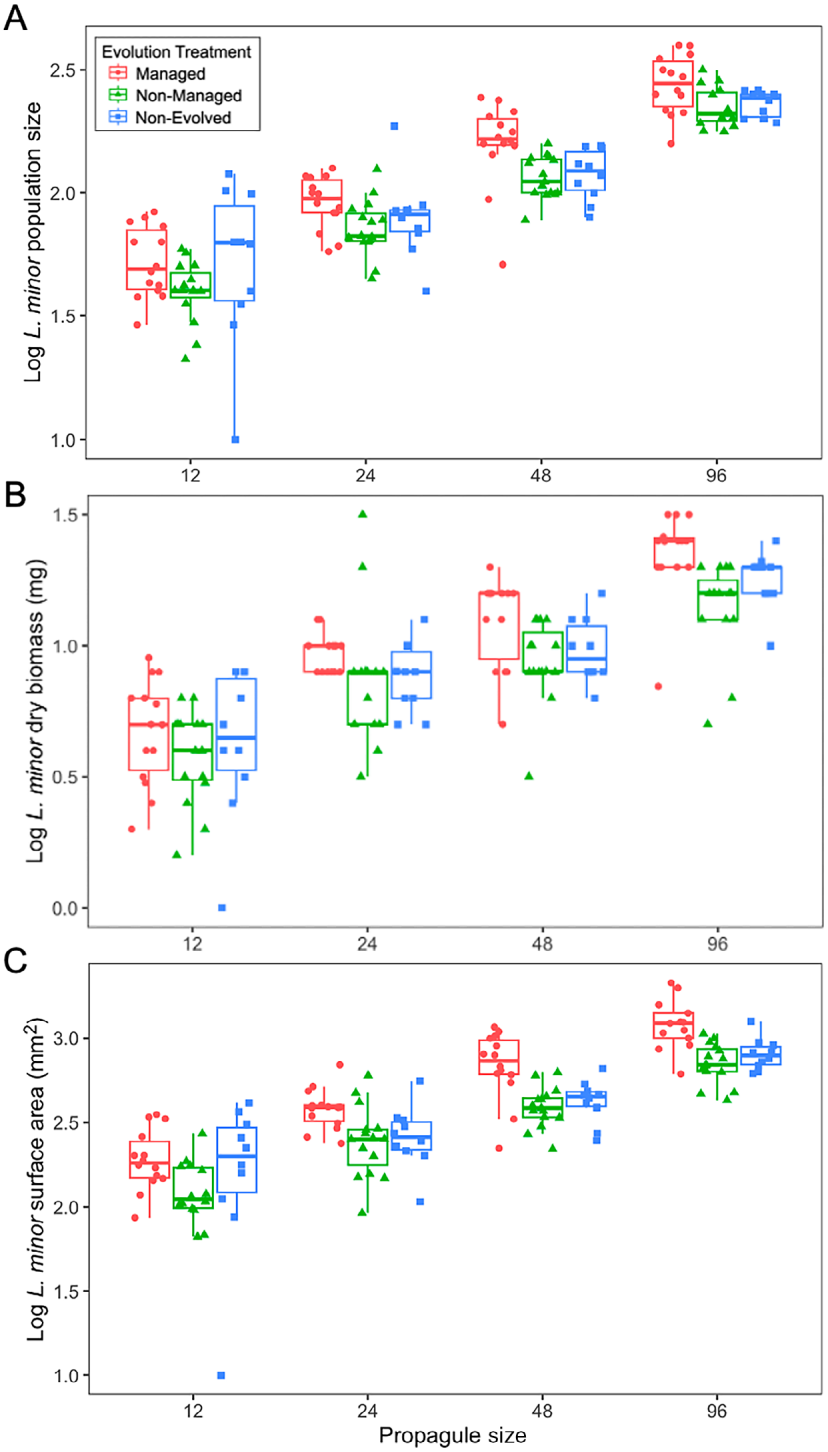
Final (A) log *L. minor* population size (B) log population biomass (mg) and (C) log population surface area (mm^2^) across evolution treatment and propagule size. Note that the *x*‐axes are graphically represented on a log_2_ scale but statistically treated as continuous.

Evolution in response to management increased the establishment of these populations growing in a resident community as measured by several metrics. When combining the logarithms of final invader abundance, biomass, and surface area together into a principal component analysis, the first principal component axis explained 92% of the variance and it was significantly affected by evolution (*F*_2,27_ = 11.658, *p* = 0.0002), propagule pressure (*F*_1,121_ = 331.232, *p* < 0.0001), but not their interaction (*F*_2,121_ = 0.280, *p* = 0.7562; Table S2, Figure S3). To gain more insight and clarity, we next present results for invader abundance, biomass, and surface area separately. First, the log final abundance after 2 weeks was significantly impacted by evolution treatment (*F*_2,27_ = 7.879, *p* = 0.0020), initial propagule size (*F*_1,123_ = 421.870, *p* < 0.0001), but not their interaction (Figure 1A; Table S3). These evolutionary differences were driven by significant differences between populations that evolved under management reaching higher abundances than the evolved non‐managed populations (higher, *p* = 0.0014). Managed populations did not significantly differ in final abundance with non‐evolved populations (*p* = 0.1044) and there were no significant differences between non‐managed and non‐evolved populations (*p* = 0.6668). Consistently, evolutionary treatment not only resulted in a numerical increase but also larger populations in terms of log final biomass (*F*_2,27_ = 8.134, *p* = 0.0017, Figure 1B) and log final surface area (*F*_2,27_ = 14.101, *p* < 0.0001, Figure 1C), which were also influenced by initial propagule size but not their interactions (Tables S4 and S5). Managed populations ended with more biomass than non‐managed (+16%, *p* = 0.0012) but not non‐evolved populations (*p* = 0.0833). Final log biomass of non‐managed and non‐evolved populations did not significantly differ (*p* = 0.3625). Surface area had a significantly greater increase for managed populations compared to non‐managed (+8.5%, *p* < 0.0001) and non‐evolved (+2%, *p* = 0.0025) populations. There was no significant difference in surface area between non‐managed and non‐evolved populations (*p* = 0.4534).

**FIGURE 2:**
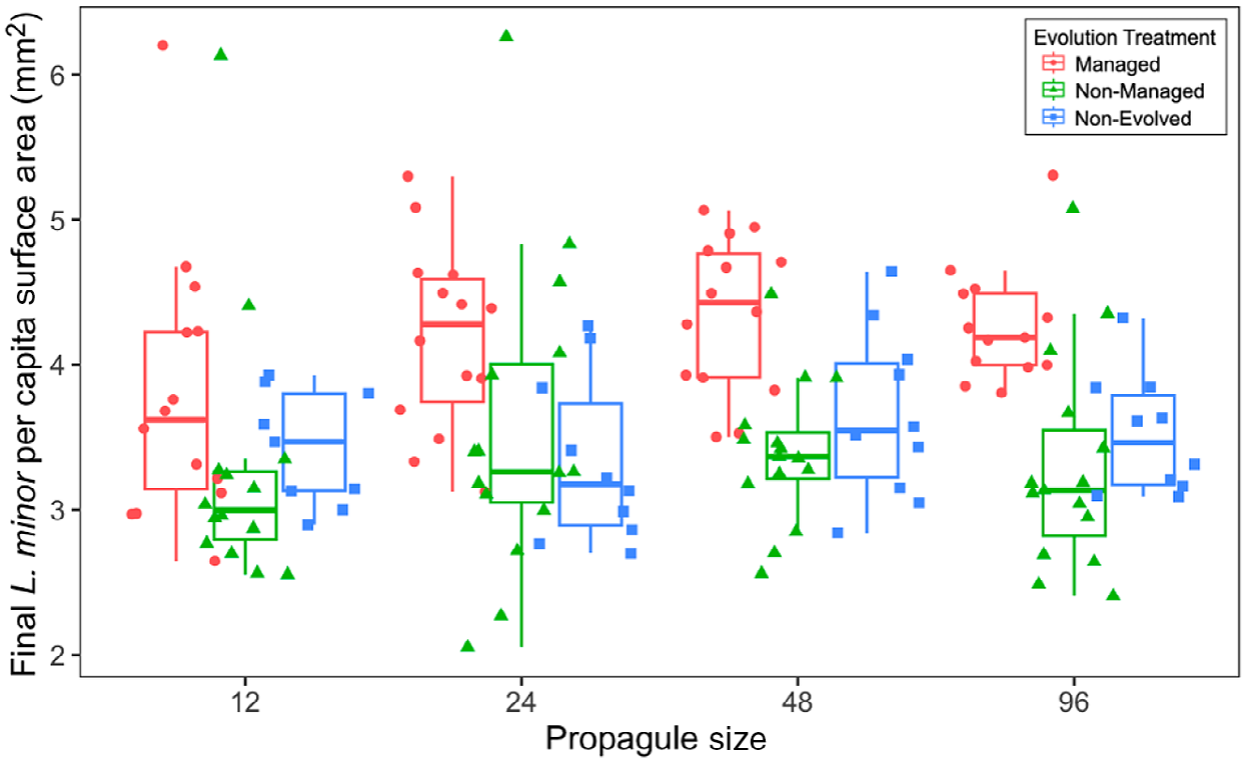
Final *L. minor* per capita surface area (mm^2^) across evolution treatment and propagule size. Note that the *x*‐axis is graphically represented on a log_2_ scale but statistically treated as continuous.

Individuals differed phenotypically at the end of the experiment but only in area. Per capita final biomass was not impacted by any treatment (*F*_2,27_ = 0.101, *p* values > 0.2, Figure S4; Table S6). Individual area was impacted only by a main effect of evolution (*F*_2,27_ = 10.120, *p* = 0.0005, Figure 2; Table S7). For individual area, populations that evolved under management were larger than non‐managed populations (15% increase in size, *p* = 0.0004). However, there was no significant difference in surface area between managed and non‐evolved populations (*p* = 0.0969) or between non‐managed and non‐evolved populations (*p* = 0.9990).

### 3.3 Evolution's Effect on *L. minor* 's Impact on Residents

**FIGURE 3:**
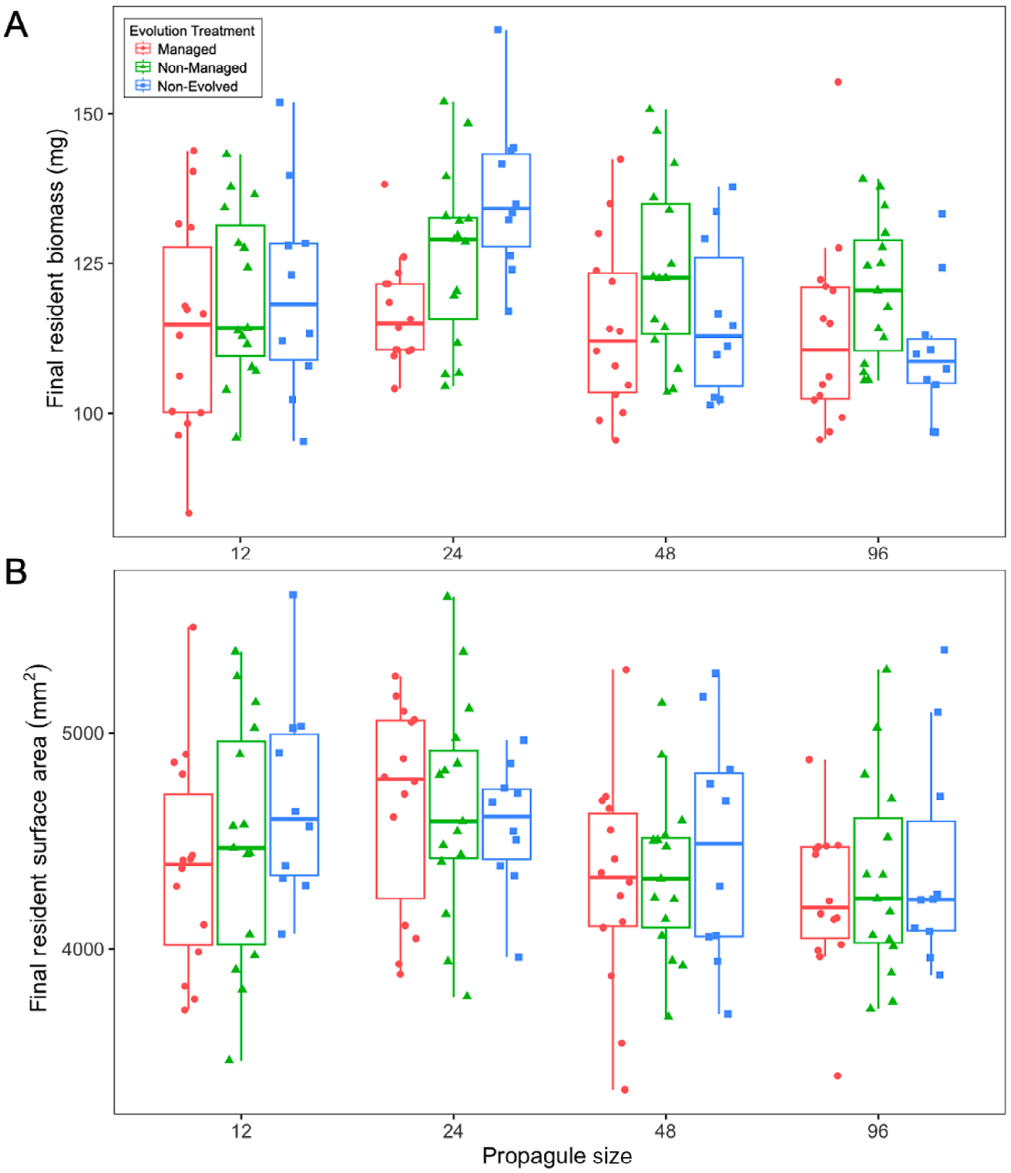
(A) Final resident biomass (mg) and (B) surface area (mm^2^) across evolution treatment and propagule size. Note that the *x*‐axes are graphically represented on a log_2_ scale but statistically treated as continuous.

While evolution might have contributed to the successful establishment of introduced populations, evolution did not alter the impact of the populations on residents among the two evolving treatments. Resident final biomass (Figure 3A) was significantly impacted by an interaction between evolution and propagule pressure (*F*_2,123_ = 3.788, *p* = 0.0253, Table S8), decreased with propagule pressure (*F*_2,123_ = 5.541, *p* = 0.0202), and there was no significant main effect of evolution (*F*_2,123_ = 1.782, *p* = 0.1875). The interaction between evolution and propagule size was driven by the non‐evolving control whose slope was in the opposite direction. For resident surface area, it was only significantly impacted by main effect of propagule size in a negative manner (*F*_1,123_ = 8.593, *p* = 0.0040, Figure 3B; Table S9).

### 3.4 Evolution's Effect on *L. minor* 's Impact on Community Size

**FIGURE 4:**
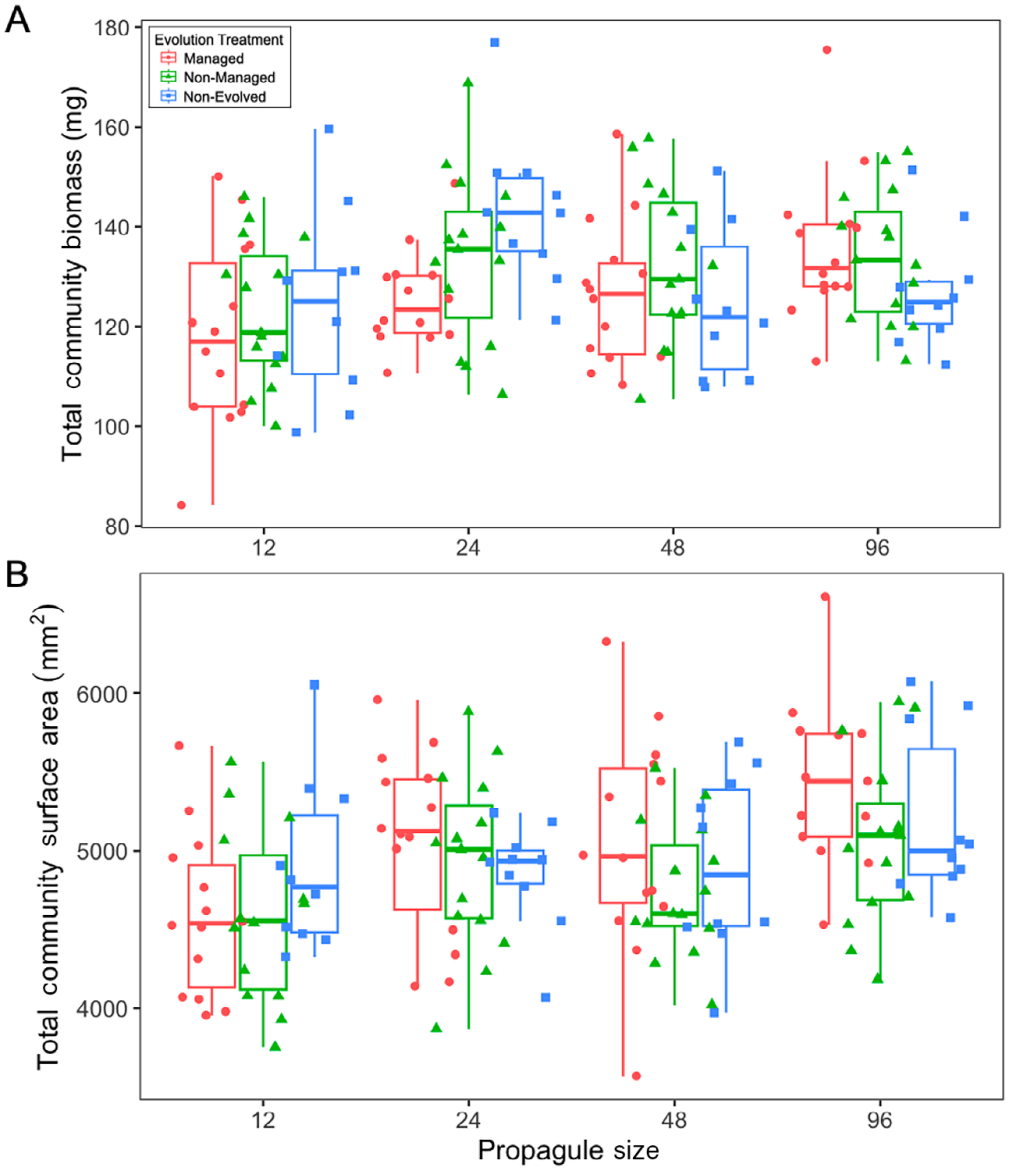
(A) Total community ( *L. minor* + residents) biomass (mg) and (B) total surface area (mm^2^) across evolution treatment and propagule size. Note that the *x*‐axes are graphically represented on a log_2_ scale but statistically treated as continuous.

There was no significant main effect of evolution alone on total community dry biomass and total community surface area (Figure 4; Tables S10 and S11). Increased propagule pressure resulted in significantly increased final community biomass (Figure 4A) and surface area (Figure 4B). However, evolution and propagule pressure significantly interacted to predict total community biomass (*F*_2,123_ = 4.235, *p* = 0.0167). With increasing propagule pressure, communities introduced with managed populations had significantly more total biomass than communities introduced with non‐evolved populations (*p* = 0.0119). While communities introduced with managed populations increased in biomass with increasing propagule size, communities introduced with non‐evolved populations on average decreased in biomass with increasing propagule size (Figure 4A). There was no significant difference in total biomass of communities introduced with managed and non‐managed populations (*p* = 0.3978) and between communities introduced with non‐managed and non‐evolved populations (*p* = 0.1849). There was also no significant interaction between evolution and propagule pressure to predict total community surface area (*F*_2,121_ = 1.354, *p* = 0.2620).

## 4 Discussion

We evolved populations with and without successive rounds of management and found that physical removal increased invasiveness but had little effect on their impact on the residents. We discuss these results and how they relate to range expansion and biological invasions, explore potential evolutionary mechanisms, and describe implications for the management of weedy or invasive species.

### 4.1 Evolution's Impact on *L. minor* Populations

With only three rounds of management, occurring over only 13–19 generations, we found that managed populations evolved the ability to invade resident communities significantly faster than non‐managed populations. The non‐evolved populations revealed the directionality of this evolutionary change. Non‐managed populations did not differ from their non‐evolved ancestors in their invasiveness, suggesting that the difference among the evolution treatments is driven by the managed populations evolving higher invasiveness as opposed to the non‐managed populations evolving reduced invasiveness.

Our result that evolution can lead to increased invasiveness is consistent with other experimental work tested in the context of range expansion. In Szűcs et al. (2017), populations evolving to a novel food source (representing a nutritional and evolutionary challenge) responded by demonstrating increased population growth and spread relative to populations where evolution was experimentally constrained. In Williams, Kendall, and Levine (2016), populations evolving to large patches in their experimental landscapes spread three times as far as their nonevolving counterparts. Conversely, in a related experiment evolving populations of *A. thaliana* in favorable environments, populations responded by decreasing their performance when exposed to stressful conditions such as drought and interspecific competition (Lustenhouwer, Williams, and Levine 2019). Taken together with our own findings, these results suggest that the evolutionary basis for invasiveness might be correlated with and selected for in disturbed and stressful environments (Lee and Gelembiuk 2008). While we did not lower the quality or disturb the environment (there were more resources per capita in managed populations versus non‐managed), the repeated culling simulated the impact of such disturbances producing a similar numerical effect on managed populations. Whether these same patterns hold true under a range of abiotic, biotic, and human induced disturbances varying in their novelty warrants further investigation.

Invasiveness increased with high propagule pressure, and although this is not surprising, it does support the idea that larger or repeated invasion events would favor invader success (Lockwood, Cassey, and Blackburn 2005). In addition, we did not observe interactions between evolution treatment and propagule size for invasiveness. This may suggest that potential sampling effects (i.e., only large propagule abundances would be a representative sample of the evolving population) are weak and that intraspecific competition among *L. minor* was not important given their rare initial abundances. In other system such mechanisms may be more impactful.

### 4.2 Evolution's Impact on Residents

Although we saw large evolutionary gains in population growth, as evidenced by increases in population abundance, mass, and area due to management, this evolution did not increase impacts on residents overall (Figure 3). This could be due to several reasons related to the ecology and evolutionary history of the *L. minor* genotypes, populations, and the residents themselves. First, it could simply be that the introduced populations were not a large enough fraction of the community or had enough time for a quantifiable impact on the residents. It is also possible that if growth conditions were more challenging (e.g., the resident community was under stress such as greater nutrient limitation or at carrying capacity) then they may have experienced more severe competitive impacts from the experimental invasions. Second, it is possible that management evolution could have impacted the relative performance of the individual resident species resulting in different resident compositions (given each species' different relative niche overlap with *L. minor* ), but we could not logistically measure each resident individually and therefore could not test this hypothesis in this experiment. For instance, *L. minor* and *S. polyrhiza* have small niche differences (Armitage and Jones 2019; Hart, Turcotte, and Levine 2019) whereas *Wolffia* spp. are morphologically very different (Landolt 1986) and could have been differentially impacted. Future experiments could explore these possibilities. Third, evolution in response to management may also cause evolution of reduced competitive impacts (trade‐off in growth rate and competitive impact) as each individual from managed populations may have, on average, a smaller impact than non‐managed individuals as they were greater in abundance (especially at 96 initial propagule size). Lastly, it is possible that a lack of an impact could be due to the fact we used a model system where our invader and recipient community share macroevolutionary histories (even if they weren't contemporarily co‐occurring). We cannot rule out the possibility that the resident community was preadapted to various *L. minor* genotypes with similar traits that evolve in our experiment and, if we used a novel invader, their impacts would have been more apparent.

Still, the overall impact of evolution on the communities was striking. The results suggest that the primary driver of evolved invasiveness was their increasing population growth rates and possibly an interaction with evolved per capita surface area (Figure S2). Communities introduced with both managed and non‐managed populations exhibited significantly increased biomass relative to communities introduced with non‐evolved communities but only at higher propagule sizes (total biomass of *L. minor* and residents, Figure 4A). This could suggest that these evolved *L. minor* populations are less directly competitive with community members resulting in increased overall yields of each invaded community. There could be evolutionary trade‐offs in traits related to population growth and interspecific competitive ability among *L. minor* populations and potentially among residents had they been given the opportunity to evolve in response to *L. minor's* presence. In an experiment testing the invasiveness and impact of evolutionarily experienced versus naïve invaders when introduced to evolutionarily experienced versus naïve residents, Faillace and Morin (2016) found that evolutionarily experienced invader performance was variable and dependent on focal species. Evolutionarily experienced residents, however, consistently decreased invasiveness of evolutionarily naïve invaders suggesting strong selection for interspecific competitive ability. Had we considered the possible synergistic or antagonistic effects of evolution in response to management and biotic competition in tandem, we might have witnessed trade‐offs in invasiveness and impactfulness (i.e., competitiveness) as the result of *L. minor* management in the presence or absence of interspecific competition. This is not wholly surprising as observational studies have found that invasiveness is often not correlated with their impacts on residents (Ricciardi and Cohen 2007).

### 4.3 Potential Evolutionary Mechanisms

One possible mechanism underlying the evolution of greater invasiveness is that management evolution selected for rapid population growth rate, at least partially through increased specific leaf area (SLA). First, we found that managed populations also evolved faster population growth rates when growing alone than the non‐managed evolved populations (Figure S1). This suggests that they differ in intrinsic growth rate which might have allowed them to invade more quickly into the resident communities. In addition, we found that managed populations consistently exhibited a 19% increase in area per frond compared to non‐managed (but not biomass per frond). This suggests that SLA is under selection during management. SLA is a leaf economic spectrum trait associate with high growth and is correlated with invasiveness in other systems (Grotkopp and Rejmánek 2007; Feng, Fu, and Zheng 2008). Although we know growth rate and SLA evolved, we do not know how other important traits, directly or indirectly under selection, evolved that might impact invasiveness such as sensitivity to and impact on interspecific competitors. Using different genotypes, Hart, Turcotte, and Levine (2019) found that *L. minor* competing with *S. polyrhiza* evolved higher growth rate and SLA but also evolved to become more sensitive to interspecific competition and did not change in competitive impact. Whether such changes occurred in our experiment due to management and whether similar changes would occur with our mixed community remains to be determined. For instance, while the evolutionary conditions under management posed greater threat of removal, those remaining experienced less competition and more resources per capita. It's possible that had we combined the synergistic effects of management and interspecific competition that we might have evolved populations possessing both high competitiveness and growth or possibly just one of the suites of traits depending on relative strength of selection of either management or competition.

### 4.4 Limitations

Our experiment and system have limitations to consider. First, given the clonal reproduction of duckweed and their very low mutation rates (Xu et al. 2019), the evolutionary changes are restricted to clonal sorting over multiple generations. While the common garden portion of this experiment lasted multiple generations, it is unknown the extent to which evolution was due to genotypic change or the persistence of long‐term epigenetic factors. Other systems may show more extensive evolution if there is sexual recombination and stronger impacts on invasiveness. We used *L. minor* as a model of an invader to gain insight into the evolution of weediness or invasiveness for several reasons including ethical and practical ones. While we believe these results help provide insight, we acknowledge that results may differ if the managed species is already a successful invader or simply if it had less evolutionary history with resident species in the community. As previously mentioned, these results might change if trade‐offs between population growth and interspecies competitive ability exist and the management evolution experiment had been carried out in communities instead of single species populations. Still, many invasive and weedy populations grow in dense monocultures where interspecific competition is weak or non‐existent. We hope more experimental evolution will be conducted on this topic in different systems.

### 4.5 Implications and Conclusions

Although our result may seem concerning for the management of weedy and invasive populations, we want to be clear that, given their potentially harmful nature, management interventions are necessary to control or reduce their impact. However, our results show that we should carefully consider how management practices may inadvertently create selective pressures that could have negative consequences on controlling problematic populations in the future. Clearly, more work is needed to determine when such concerns are warranted. Whether our results are general to other forms of management (herbicides, biocontrol agents, etc.) remains to be tested. Our results demonstrate that future tests of adaptive evolution to pest management strategies should conduct trials in appropriate ecological settings particularly following the removal of selective agents. Researchers and managers might intentionally vary the frequency and intensity of management strategies to assess potential eco‐evolutionary impacts before deploying them at scale. It is likely that an integrative management approach using multiple control strategies with different mechanisms of action may be needed to avoid or at least delay evolutionary responses leading to increasingly negative ecological consequences as is the case with other types of pests (Brown 2015; Turcotte et al. 2017). Given the possible eco‐evolutionary consequences, greater emphasis could be placed on the containment of populations facing management, preventing their further introduction and spread into new communities.

To understand, mitigate, and manage emerging weedy and invasive populations, our results suggest that we should not ignore rapid evolution and its interactions with other ecological processes. A new generation of studies that experimentally assess evolution's impact may help avoid unforeseen consequences of short‐ and long‐term pest management strategies.

## Conflicts of Interest

The authors declare no conflicts of interest.

## Supporting information


Data S1.

## Data Availability

Data for this study are available at the Dryad Digital Repository: https://datadryad.org/stash/share/90suHKJaNGYO8ZIjz39ABPFio74wJuKGlE4fb9Z9vHs.
