# Supplementary material for: Evolution in Response to Management Increases Invasiveness Among Experimental Populations of Duckweed ( Lemna minor )
Source: Evol Appl. 2024 Dec 26;17(12):e70060. doi: 10.1111/eva.70060 (PMC11671222; doi:10.1111/eva.70060)

**Supplementary material**

**Table S1** - Sampling location for *Lemna minor* genotypes and resident species.

| **Genotype** | **Location** | **County** | **Latitude** | **Longitude** |
| --- | --- | --- | --- | --- |
| LM.00013 | Buhl Park Big Pond | Mercer | 41.24783333 | -80.48428333 |
| LM.00027 | Cross Creek State Park | Washington | 40.25856667 | -80.40021667 |
| LM.00039 | Deer Lakes Park Pond One | Allegheny | 40.61974 | -79.82857 |
| LM.00044 | Deer Lakes Park Pond Two | Allegheny | 40.62206667 | -79.82441667 |
| LM.00090 | Keystone Park Keystone Lake | Westmoreland | 40.37385 | -79.37743333 |
| LM.00096 | Mingo Creek Office Trail | Washington | 40.18341667 | -80.03751667 |
| LM.00110 | Moraine State Park Lake Arthur | Butler | 40.97156667 | -80.0186 |
| LM.00121 | Northmoreland Park Pond | Westmoreland | 40.5764 | -79.61638333 |
| LM.00139 | Pymatuning Jack's Pond | Crawford | 41.67505 | -80.51276667 |
| LM.00145 | Peter's Lake Park Peter's Lake | Washington | 40.25846667 | -80.11083333 |
| LM.00195 | Schenley Park Westinghouse Pond | Allegheny | 40.4393 | -79.94296667 |
| LM.00236 | Rutherglen Road Pond | Allegheny | 40.3838 | -80.17563333 |
| Resident species | PA State Gamelands 151 | Mercer | 41.106241 | -80.134670 |

*L. minor* *establishment*

**Table S2** – ANOVA table for linear mixed effects model using evolution treatment, propagule pressure, and their interactions as predictors for *L. minor* composite values for PC1 scores (aggregated from log final abundance, log biomass, and log surface area). Two images were low quality, thus reducing sample size.

|  | **Numerator DF** | **Denominator DF** | **F-value** | **p-value** |
| --- | --- | --- | --- | --- |
| (Intercept) | 1 | 121 | 0.0000 | 1.0000 |
| Evolution | 2 | 27 | 11.6582 | 0.0002 |
| Propagule Pressure | 1 | 121 | 331.2324 | <.0001 |
| Interaction | 2 | 121 | 0.2801 | 0.7562 |

**Table S3** – ANOVA table for linear mixed effects for log final *L. minor* abundance.

|  | **Numerator DF** | **Denominator DF** | **F-value** | **p-value** |
| --- | --- | --- | --- | --- |
| (Intercept) | 1 | 123 | 26270.069 | <.0001 |
| Evolution | 2 | 27 | 7.879 | 0.0020 |
| Propagule Pressure | 1 | 123 | 421.870 | <.0001 |
| Interaction | 2 | 123 | 0.407 | 0.6668 |

**Table S4** – ANOVA table for linear mixed effects for log *L. minor* biomass.

|  | **Numerator DF** | **Denominator DF** | **F-value** | **p-value** |
| --- | --- | --- | --- | --- |
| (Intercept) | 1 | 123 | 3795.952 | <.0001 |
| Evolution | 2 | 27 | 8.134 | 0.0017 |
| Propagule Size | 1 | 123 | 186.148 | <.0001 |
| Interaction | 2 | 123 | 0.723 | 0.4876 |

**Table S5** – ANOVA table for linear mixed effects model using evolution treatment, propagule pressure, and their interactions as predictors for log *L. minor* surface area. Two images were low quality, thus reducing sample size.

|  | **Numerator DF** | **Denominator DF** | **F-value** | **p-value** |
| --- | --- | --- | --- | --- |
| (Intercept) | 1 | 121 | 23278.163 | <.0001 |
| Evolution | 2 | 27 | 14.101 | 0.0001 |
| Propagule Size | 1 | 121 | 251.760 | <.0001 |
| Interaction | 2 | 121 | 0.206 | 0.8142 |

**Table S6** – ANOVA table for linear mixed effects model using evolution treatment, propagule pressure, and their interactions as predictors for *L. minor* per capita biomass.

|  | **Numerator DF** | **Denominator DF** | **F-value** | **p-value** |
| --- | --- | --- | --- | --- |
| (Intercept) | 1 | 123 | 265.80276 | <.0001 |
| Evolution | 2 | 27 | 0.14742 | 0.9038 |
| Propagule Size | 1 | 123 | 1.25697 | 0.2100 |
| Interaction | 2 | 123 | 1.50123 | 0.2752 |

**Table S7** – ANOVA table for linear mixed effects model using evolution treatment, propagule pressure, and their interactions as predictors for *L. minor* per capita surface area. Two images were low quality, thus reducing sample size.

|  | **Numerator DF** | **Denominator DF** | **F-value** | **p-value** |
| --- | --- | --- | --- | --- |
| (Intercept) | 1 | 121 | 1957.8826 | <.0001 |
| Evolution | 2 | 27 | 10.2657 | 0.0005 |
| Propagule Size | 1 | 121 | 1.6903 | 0.1960 |
| Interaction | 2 | 121 | 1.2105 | 0.3016 |

*L. minor* *impact on residents*

**Table S8** – ANOVA table for linear mixed effects model using evolution treatment, propagule pressure, and their interactions as predictors for resident biomass.

|  | **Numerator DF** | **Denominator DF** | **F-value** | **p-value** |
| --- | --- | --- | --- | --- |
| (Intercept) | 1 | 123 | 3196.747 | <.0001 |
| Evolution | 2 | 27 | 1.782 | 0.1875 |
| Propagule Size | 1 | 123 | 5.541 | 0.0202 |
| Interaction | 2 | 123 | 3.788 | 0.0253 |

**Table S9** – ANOVA table for linear mixed effects model using evolution treatment, propagule pressure, and their interactions as predictors for resident surface area.

|  | **Numerator DF** | **Denominator DF** | **F-value** | **p-value** |
| --- | --- | --- | --- | --- |
| (Intercept) | 1 | 123 | 6829.469 | <.0001 |
| Evolution | 2 | 27 | 0.253 | 0.7787 |
| Propagule Size | 1 | 123 | 8.593 | 0.0040 |
| Interaction | 2 | 123 | 0.010 | 0.9896 |

*Community change*

**Table S10** – ANOVA table for linear mixed effects model using evolution treatment, propagule pressure, and their interactions as predictors for total community biomass.

|  | **Numerator DF** | **Denominator DF** | **F-value** | **p-value** |
| --- | --- | --- | --- | --- |
| (Intercept) | 1 | 123 | 3669.755 | <.0001 |
| Evolution | 2 | 27 | 0.480 | 0.6241 |
| Propagule Size | 1 | 123 | 7.029 | 0.0091 |
| Interaction | 2 | 123 | 4.235 | 0.0167 |

**Table S11** – ANOVA table for linear mixed effects model using evolution treatment, propagule pressure, and their interactions as predictors for total community surface area. Two images were lost thus reducing sample size.

|  | **Numerator DF** | **Denominator DF** | **F-value** | **p-value** |
| --- | --- | --- | --- | --- |
| (Intercept) | 1 | 121 | 6531.074 | <.0001 |
| Evolution | 2 | 27 | 1.171 | 0.3252 |
| Propagule Size | 1 | 121 | 19.549 | <.0001 |
| Interaction | 2 | 121 | 1.354 | 0.2620 |

**Figure S1** – Final *L. minor* population A) abundance B) biomass (mg) and C) surface area (mm^2^) when growing from a population size of 24 individuals in absence of residents.


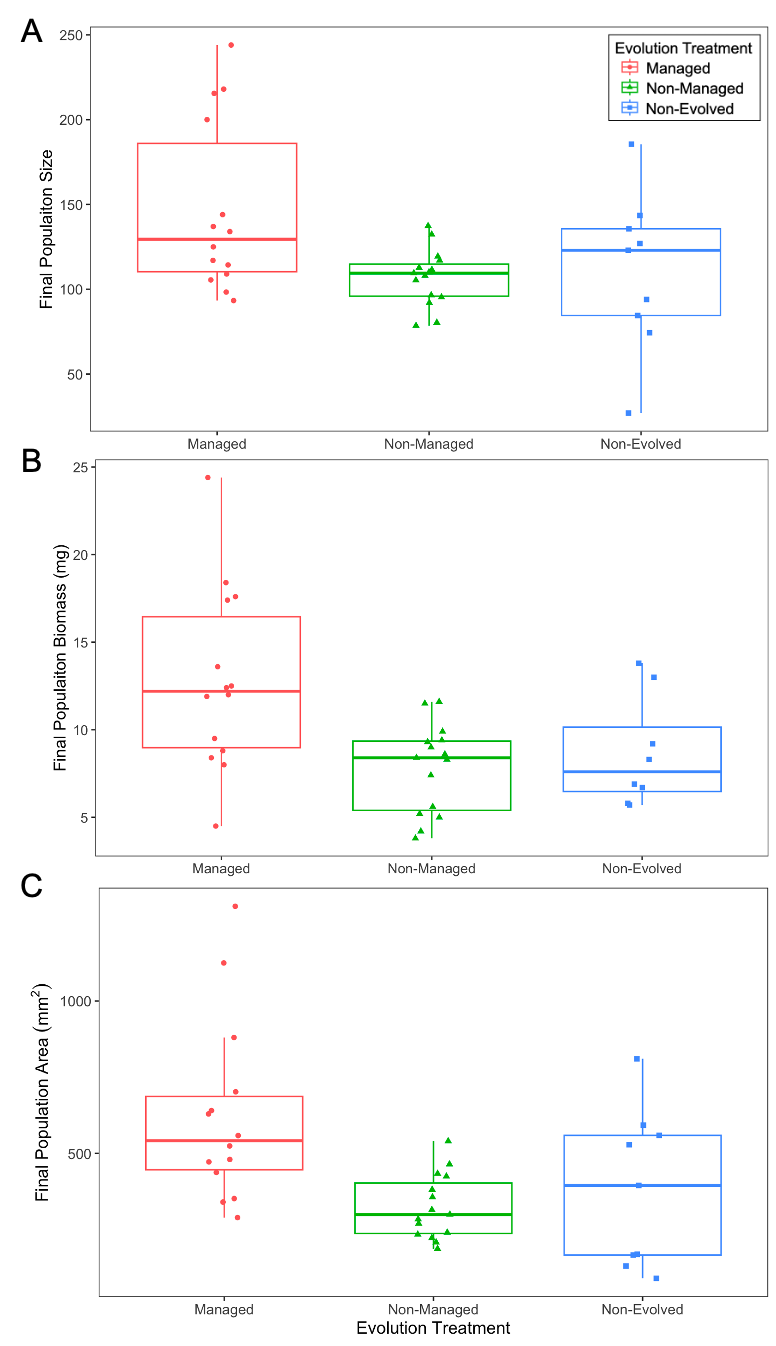


**Figure S2** – Final *L. minor* per capita A) biomass (mg) and B) surface area (mm^2^) when growing from an initial population size of 24 individuals in absence of residents.


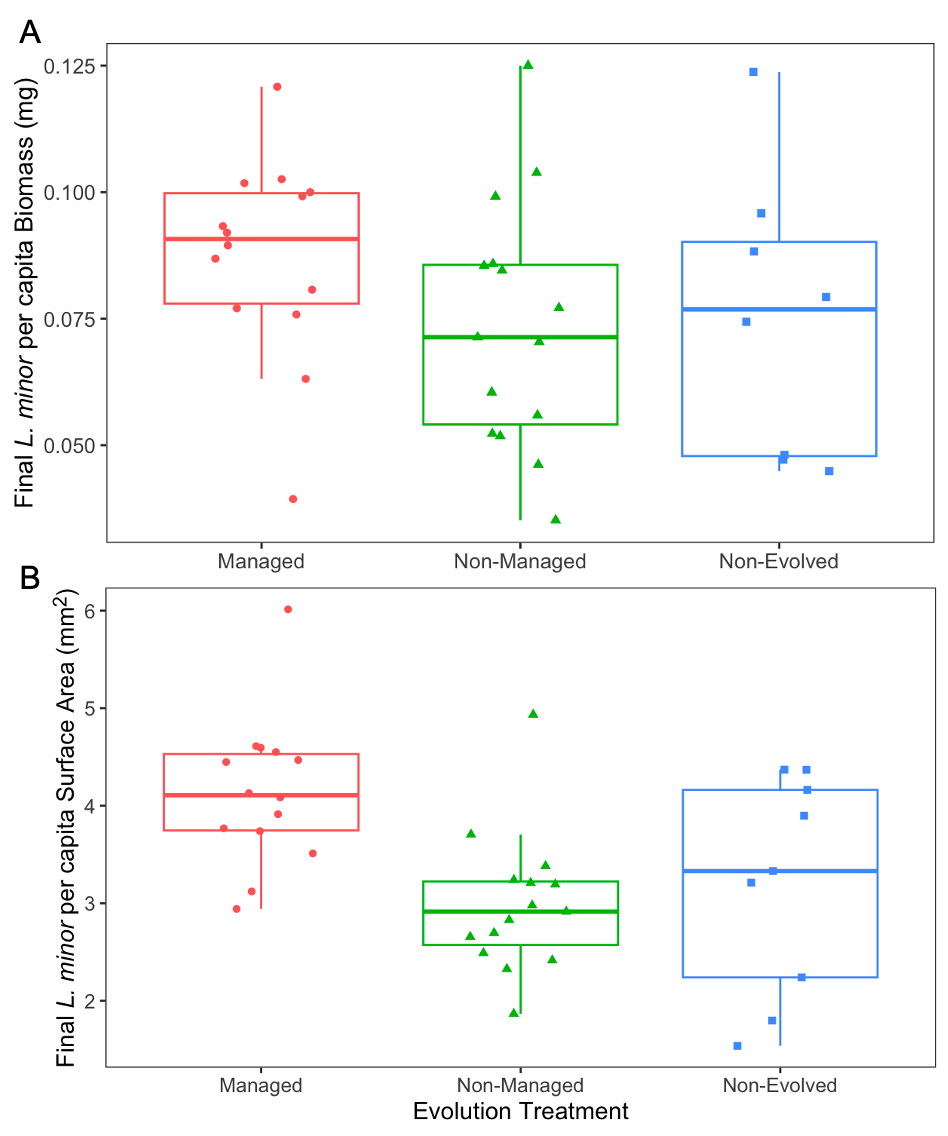


**Figure S3** – Principal component axis 1 (PC1) as a composite for log final abundance, log biomass, and log surface area of *L. minor* given evolution treatment and propagule size. Note that the x-axis is graphically represented on a log_2_ scale but statistically treated as continuous.

**
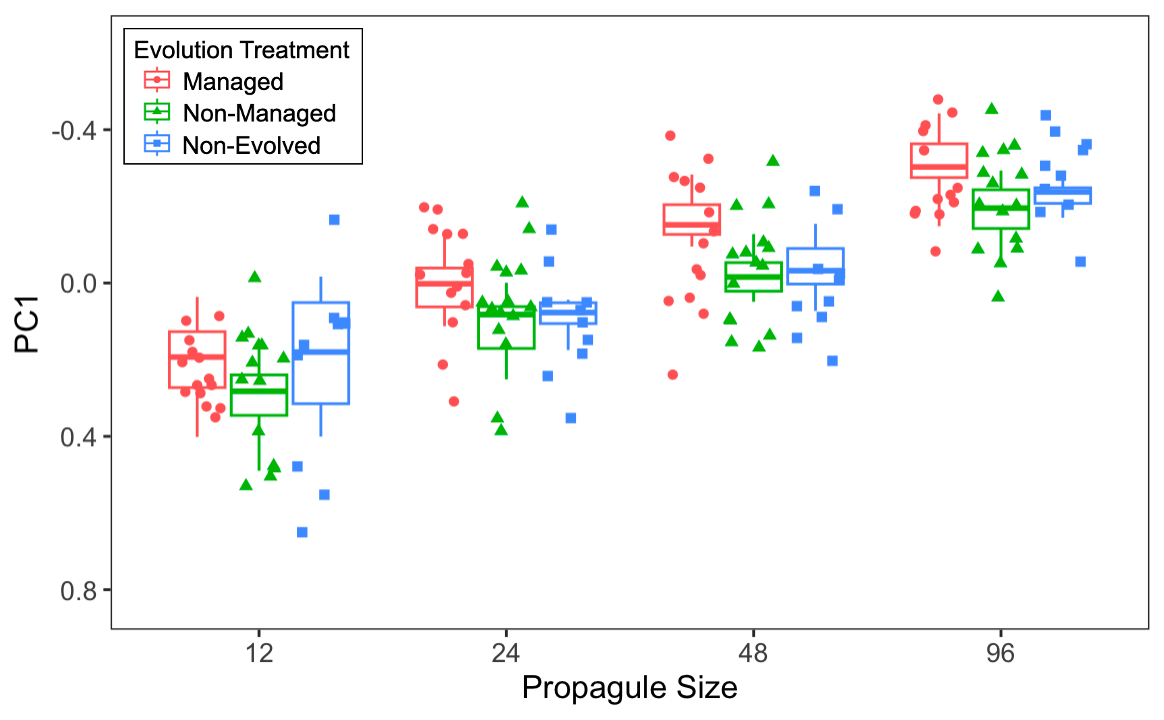
**

**Figure S4** – Final per capita biomass (mg) given evolution treatment and propagule size. Note that the x-axis is graphically represented on a log_2_ scale but statistically treated as continuous.


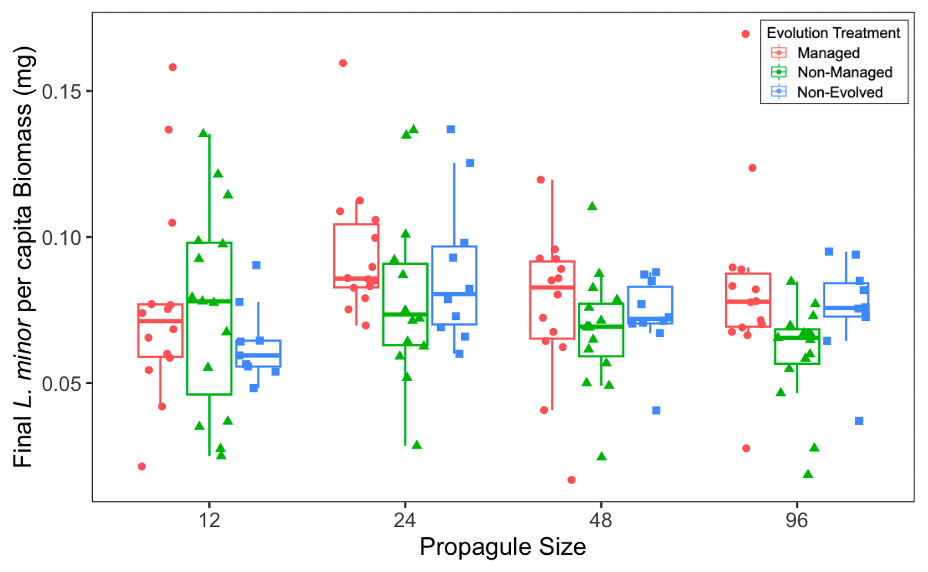

Supplement: Supplementary file 1 — Data S1. [file EVA-17-e70060-s001.docx]
